# Immunophenotyping of rheumatoid arthritis reveals a linkage between HLA-DRB1 genotype, CXCR4 expression on memory CD4^+^ T cells, and disease activity

**DOI:** 10.1038/srep29338

**Published:** 2016-07-07

**Authors:** Yasuo Nagafuchi, Hirofumi Shoda, Shuji Sumitomo, Shinichiro Nakachi, Rika Kato, Yumi Tsuchida, Haruka Tsuchiya, Keiichi Sakurai, Norio Hanata, Shoko Tateishi, Hiroko Kanda, Kazuyoshi Ishigaki, Yukinori Okada, Akari Suzuki, Yuta Kochi, Keishi Fujio, Kazuhiko Yamamoto

**Affiliations:** 1 Department of Allergy and Rheumatology, Graduate School of Medicine, The University of Tokyo, Tokyo, Japan; 2 Laboratory for Autoimmune Diseases, Center for Integrative Medical Sciences, RIKEN, Yokohama, Japan; 3 Department of Statistical Genetics, Osaka University Graduate School of Medicine, Osaka, Japan

## Abstract

Rheumatoid arthritis (RA) is a chronic autoimmune inflammatory disease that leads to destructive arthritis. Although the HLA class II locus is the strongest genetic risk factor for rheumatoid arthritis, the relationship between HLA class II alleles and lymphocyte activation remains unclear. We performed immunophenotyping of peripheral blood mononuclear cells on 91 HLA-DRB1-genotyped RA patients and 110 healthy donors. The frequency of memory CXCR4^+^CD4^+^ T cells, and not Th1 and Th17 cells, was significantly associated with disease severity by multiple linear regression analysis. RA patients with one or more susceptible HLA-DR haplotypes (shared epitope: SE) displayed a significantly higher frequency of memory CXCR4^+^CD4^+^ T cells. Moreover, the frequency of memory CXCR4^+^CD4^+^ T cells significantly correlated with the expression level of HLA-DR on B cells, which was elevated in RA patients with SE. *In vitro* analysis and transcriptomic pathway analysis suggested that the interaction between HLA-DR and T cell receptors is an important regulator of memory CXCR4^+^CD4^+^ T cells. Clinically, a higher frequency of memory CXCR4^+^CD4^+^ T cells predicted a better response to CTLA4-Ig. Memory CXCR4^+^CD4^+^ T cells may serve as a powerful biomarker for unraveling the linkage between HLA-DRB1 genotype and disease activity in RA.

Rheumatoid arthritis (RA) is a chronic autoimmune inflammatory disease that leads to destructive arthritis. Both genetic and environmental factors contribute to RA pathogenesis^1^. A recent meta-analysis of genome-wide association studies identified as many as 101 RA risk loci^2^. In particular, the HLA-DRB1 genotype was the first identified and by far the strongest genetic risk factor for RA^3^,^4^. The shared epitope (SE), a common amino acid sequence at positions 70–74 of HLA-DRB1, is recognized for its association with anti-cyclic citrullinated peptide antibody (ACPA)-positive RA^5^. It is thought that citrullinated autoantigen epitopes bind to HLA-DRB1 that contain the SE and are presented to CD4^+^ T cells, which contribute to autoimmunity^6^. Moreover, SE is an important risk factor for severe bone destructive disease^5^,^7^.

Nevertheless, in spite of tremendous efforts to identify immunological abnormalities in RA, few studies have identified any linkage between SE and adaptive immunity. To understand the immunological role of SE, immune cell populations associated with SE should be identified. The key role of CD4^+^ T cells in RA pathogenesis is highlighted by the fact that RA genetic risk loci preferentially map to enhancers and promoters active in CD4^+^ T cell subsets^8^. Standardized human immunophenotyping has been proposed for classifying CD4^+^ T cells into conventional Th1, Th2, and Th17 cell types based on their expression of the chemokine receptors CXCR3 and CCR6^9^. Although a number of researchers have examined the frequency of Th1, Th2, Th17, Tfh, and Treg cells in RA, these populations show no clear association with RA disease activity measures, such as Disease Activity Score 28 joints-ESR (DAS28esr) and Health Assessment Questionnaire Disability Index (HAQ)^10^,^11^,^12^,^13^. Therefore, other markers for CD4^+^ T cells need to be investigated.

In the RA synovium, there are ectopic lymphoid follicles as well as clonally expanded T cells and antigen-specific B cells that recognize citrullinated autoantigens^14^,^15^. These findings strongly suggest that acquired immunity against autoantigens promotes local inflammation in the synovium, such as macrophage activation and inflammatory cytokine production, including TNF-α and IL-6. The chemokine receptor CXCR4 plays a central role in the homing and retention of CD4^+^ T cells^16^,^17^. The CXCR4 ligand CXCL12 (also known as SDF-1) and the recently identified ligand macrophage migration inhibitory factor (MIF) are both produced by synovial fibroblasts and are increased in RA synovium^18^,^19^,^20^. It has also been reported that inflammatory cytokine-activated CD4^+^ T cells express high levels of CXCR4^21^ and that T-cell-specific CXCR4-deficient mice show a dramatic decrease in the incidence of arthritis^22^.

Based on these preceding reports, we attempted to identify lymphocyte subsets that are associated with HLA-DRB1 and RA disease activity. We analyzed HLA-DRB1-genotyped RA patients by 24-subset immunophenotyping combined with CXCR4 expression, HLA-DR quantification on antigen-presenting cells, and multiplex serum cytokine analysis.

## Results

### Study populations

We recruited 91 RA patients and 110 healthy donors (HD) (Table S1). 61 RA patients with at least one HLA-DRB1 SE allele were considered to be SE-positive RA (SE + RA). Among the SE + RA group, 44 patients (72%) had at least one HLA-DRB1 04:05 allele, 14 patients (23%) had at least one 01:01 allele, and 6 patients (10%) had the 04:01 allele. The SE + RA and SE-negative RA (SE-RA) groups showed comparable baseline characteristics, including rheumatoid factor (RF) titer, DAS28esr disease activity score, and HAQ functional disability index. ACPA titer was significantly higher in the SE + RA group compared to the SE-RA group, as reported^5^.

### Memory CD4^+^ T cells are associated with ACPA and SE positivity in RA

**Figure 1:**
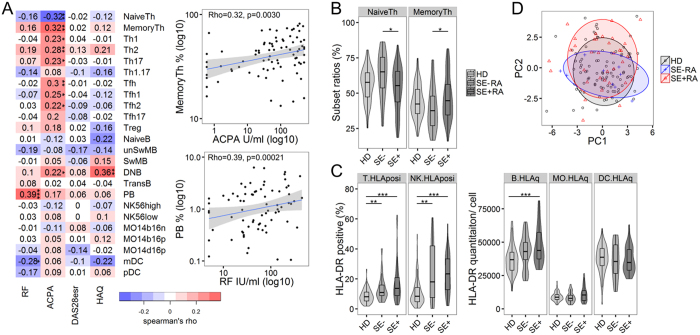
Memory CD4^+^ T cells are associated with ACPA and SE positivity in RA. (**A**) Heatmap of correlation matrix between 24 immunophenotyped subsets and four clinical parameters: RF, ACPA, DAS28esr and HAQ in RA patients (n = 91). Representative scatter plots and linear regression line with 95% confidence intervals are shown on the right. See Table S2 for subset definitions. (**B,C**) Comparison of NaiveTh and MemoryTh subset ratios and HLA-DR expression on T cells, NK cells, B cells, monocytes (MO), and dendritic cells (DC) between healthy donors (HD), shared epitope (SE)-negative RA patients, and SE-positive RA patients (HD; n = 110, SE-RA; n = 30, SE + RA; n = 61). For T cells and NK cells analyses, HLA-DR-positive ratios are used. B cells, MO, and DC analyses are based on flow cytometric HLA-DR quantitative expression per cells. (**D**) Principal component analysis of 24 immunophenotyped subsets to summarize the differences between HD, SE-RA, and SE + RA. PC1 explained 24% and PC2 explained 14% of the total variance (HD; n = 93, SE-RA; n = 10, SE + RA; n = 35). *p < 0.05 **p < 0.01 ***P < 0.001 Spearman’s tests (**A**) or Kruskal-Wallis tests with post-hoc Wilcoxon tests with Bonferroni corrections (**B,C**).

We performed flow cytometric 24-subset immunophenotyping on freshly isolated PBMC in order to assess global immunological changes in RA patients (Table S2, Figure S1). We compared different cell subset frequencies with clinical parameters (RF, ACPA, DAS28esr, and HAQ) in order to identify cell subsets that are associated with clinical sequelae. The percentage of conventional CD4^+^ T cell subsets, including Th1 and Th17 cells, showed no association with disease activity and HAQ, although the percentage of memory CD4^+^ T cells (MemoryTh) and ACPA displayed a weak correlation (Fig. 1A). Notably, the MemoryTh ratio was significantly increased in SE + RA (Fig. 1B). These results suggest that memory CD4^+^ T cells are associated with the production of ACPA and SE positivity. With regard to B cells, the percentages of plasmablasts (PB) correlated with RF titer (Fig. 1A).

Comparisons between HD and RA revealed immunological abnormalities in all of the major cell populations studied: CD4^+^ T cells, B cells, NK cells, monocytes, and DC (Figure S2). The increased frequency of HLA-DR-positive T cells and NK cells indicates that these populations are activated in RA patients (Fig. 1C). Furthermore, B cells from SE + RA patients had significantly higher expression of HLA-DR compared to B cells from HD, while monocytes and dendritic cells exhibited similar HLA-DR levels (Fig. 1C). There was no significant difference between SE-HD and SE + HD in HLA-DR expression on B cells (Figure S3). Increased HLA class II and HLA-DR molecules on RA B cells have been reported^23^,^24^. The increased HLA-DR expression on SE + RA B cells suggests that B cells may be important for antigen presentation in RA. However, principal component analysis (PCA) of our immunophenotyping data revealed global similarity between HD, SE-RA, and SE + RA patients (Fig. 1D), indicating that conventional immunophenotyping is not specific enough to distinguish between them.

### RF and inflammatory cytokines

Correlation analysis of serum cytokine levels showed that RF titer strongly correlated with serum inflammatory cytokines, such as IL-1β, IL-21, IFN-γ, and IL-17A (Figure S4A). Soluble gp130 (sgp130), which neutralizes IL-6 and soluble IL-6 receptor (sIL-6R) complex^25^, showed a strong negative correlation with RF (Figure S4A). Serum cytokines were not correlated with DAS28esr. Among the cytokines tested, only IL-10 displayed a moderate correlation for HAQ (Rho = 0.44, p = 0.027). The concentrations of multiple inflammatory cytokines were increased in RA, revealing a clear global difference between HD and RA (Figure S4B,C). These data indicate that the inflammatory cytokine environment in RA correlated best with RF, and not ACPA and DAS28esr.

### CXCR4 expression is increased on SE + RA memory CD4^+^ T cells

**Figure 2:**
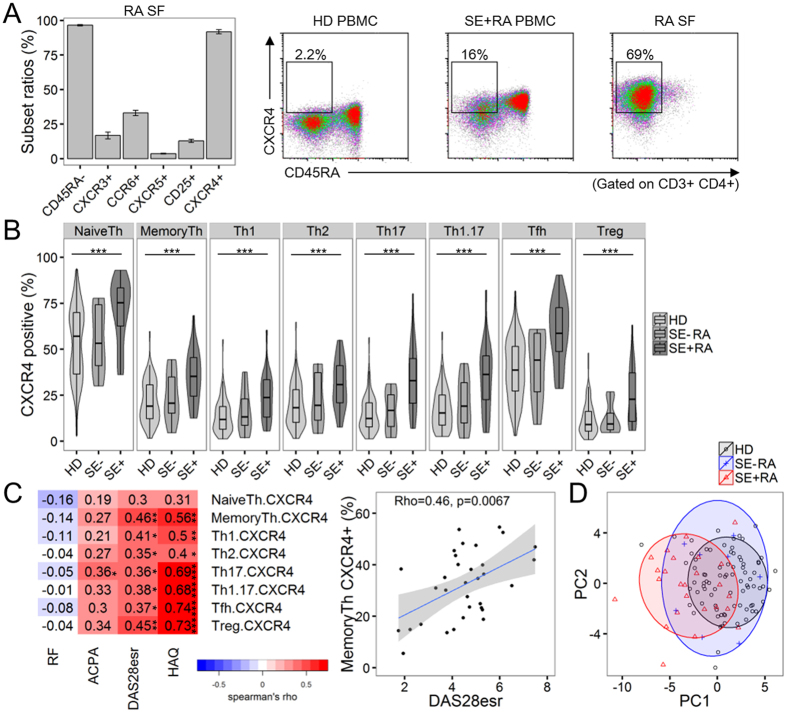
CXCR4 expression on memory CD4^+^ T cells is correlated with disease activity. (**A**) CXCR4 expression on synovial fluid (SF) and PBMC CD4^+^ T cells. Left: subset ratios of memory (CD45RA-), Th1 (CXCR3), Th17 (CCR6), Tfh (CXCR5), Treg (CD25) and CXCR4 + RA synovial fluid CD4^+^ T cells (n = 5). Mean ± SEM values. Right: representative plots of CXCR4 expression in PBMC and synovial fluid (SF) samples from healthy donors (HD) and RA patients. The CD45RA^−^CXCR4^+^ ratios are shown in the plots. The CXCR4 positivity of the CD45RA- subset (MemoryTh subset) is HD 7.8%, SE + RA 36%, and SE + RA 87% in the representative plots. (**B**) Comparison of CXCR4 expression on CD4^+^ T cell subsets among healthy donors (HD), shared epitope (SE)-negative RA patients, and SE-positive RA patients (HD; n = 82, SE-RA; n = 8, SE + RA; n = 27). (**C**) Correlations between CXCR4 expression on CD4^+^ T cell subsets and clinical parameters in RA patients (n = 35). A Representative scatter plot is shown on the right, as in Figure 1A. Tfh1, Tfh2, and Tfh17 subpopulations were eliminated from the analysis (**B,C**). (**D**) Principal component analysis of immunophenotyped subsets and CXCR4 expression on CD4^+^ T cell subsets to summarize the differences between HD, SE-RA, and SE + RA. PC1 explained 29% and PC2 explained 16% of the total variance (HD; n = 81, SE-RA; n = 8, SE + RA; n = 25). *p < 0.05 **p < 0.01 ***P < 0.001 Spearman’s test (**C**) or Kruskal-Wallis tests with post-hoc Wilcoxon tests with Bonferroni corrections (**B**).

Immunophenotyping of conventional lymphocyte populations and cytokine measurements did not reveal any factors significantly associated with RA disease activity. In accordance with this observation, the expression of Th1, Th17, Tfh, and Treg markers on synovial CD4^+^ T cells is relatively limited (Fig. 2A). In contrast, most synovial CD4^+^ T cells expressed CXCR4, as previously reported^16^. Although only a limited fraction of memory CD4^+^ T cells expressed CXCR4 in HD, both memory and naive CD4^+^PBMC T cells showed significantly enhanced CXCR4 expression, especially in SE + RA patients (Fig. 2A,B). There was no significant difference between SE-HD and SE + HD in CXCR4 expression on memory CD4^+^ T cells (Figure S5). Additionally, enhanced CXCR4 expression was observed in RA patients with high ACPA titers, which also corresponded to SE + RA (Figure S6). CXCR4 expression on various memory CD4^+^ T cell subsets, such as Tfh or Treg cells, showed moderate to strong correlations with DAS28esr and HAQ (Fig. 2C). In particular, CXCR4 expression on memory CD4^+^ T cells (MemoryTh), which includes the Th1, Th17, Tfh, and Treg subsets, was associated with DAS28esr in a multiple linear regression model that included CXCR4 expression on various CD4^+^ T cell subsets (Table S3). Also, CXCR4 expression on memory CD4^+^ T cells, not the frequency of conventional CD4^+^ T cell subsets, was associated with DAS28esr by multiple linear regression analysis (Table 1). Similar results were obtained with LASSO regression, a penalized regression model. Although steroid exposure has been reported to be associated with CXCR4 expression^26^, CXCR4 expression on memory CD4^+^ T cells was not significantly correlated with treatment drug (PSL or MTX) dose levels in RA. It is possible that treatment drugs might have some effects on CXCR4 expression on CD4^+^ T cells. The enhanced CXCR4 expression on various helper T cell subsets suggested the presence of generalized immunological modifications on memory CD4^+^ T cells in RA. When CXCR4 expression on CD4^+^ T cell subsets was combined with conventional immunophenotyping, we were able to detect a significant difference between HD and SE + RA by PCA, indicating that CXCR4 expression on CD4^+^ T cells is a key feature discriminating HD and SE + RA (Figs 1D and 2D).

CXCR4 is widely expressed on multiple immune cells, such as B cells, monocytes, and neutrophils^27^. In addition to CD4^+^ T cells, we examined CXCR4 expression on B cell subsets. Of the B cell subsets, only unswitched memory B cells from SE + RA had enhanced CXCR4 expression (Figure S7). Further research is needed to examine the role of SE in CXCR4 expression on innate immune cells. We also analyzed the expression of CCR7, a marker of central memory CD4^+^ T cells^9^. There were no significant differences in CCR7 expression on CD4^+^ T cells between HD, SE-RA, and SE + RA (Figure S8). These results also highlight the significance of enhanced CXCR4 expression on SE + RA CD4^+^ T cells.

### CXCR4 expression is regulated by cytokines and HLA-DR

**Figure 3:**
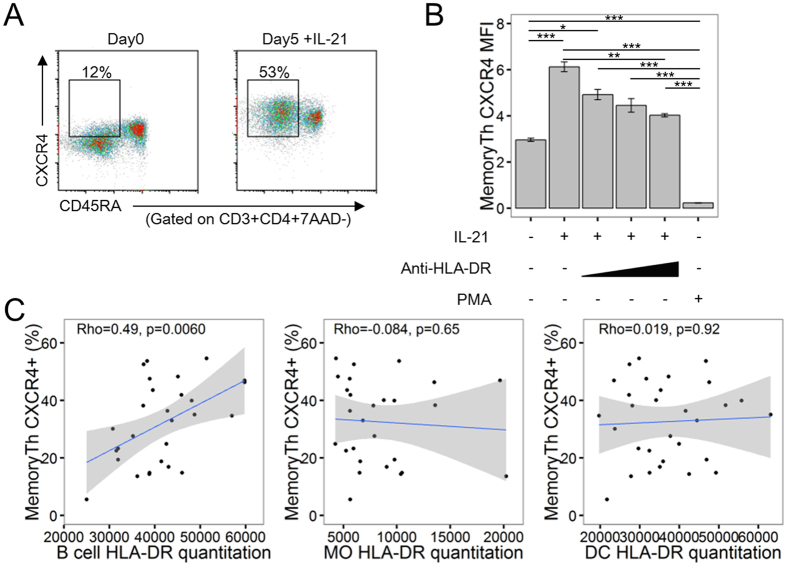
CXCR4 expression on memory CD4^+^ T cells is regulated by IL-21 and HLA-DR on B cells. (**A,B**) HD PBMCs were cultured for 5 days under the indicated conditions. (**A**) Representative plots of CXCR4 expression from Day 0 and Day 5 PBMC. (**B**) Day 5 CXCR4 mean fluorescence intensity (MFI) on memory CD4^+^ T cell subsets in each condition. Mean ± SEM values. Results are representative of two independent experiments. n = 4 per group. (**C**) Scatter plots of B cell, monocyte (MO), and dendritic cell (DC) HLA-DR quantitative expression value per cells and CXCR4-positive ratios of the MemoryTh subset in RA patients (n = 35). *p < 0.05 **p < 0.01 ***P < 0.001 Unpaired t-tests with Bonferroni corrections.

Previous reports have demonstrated that CXCR4 expression on CD4^+^ T cells is regulated by common gamma chain family cytokines, such as IL-2, IL-4, IL-7, and IL-15^28^. IL-21 is also a member of the common gamma chain family cytokines and was elevated in the serum of RA patients (Figure S4B). It has been reported that IL-21 is an important cytokine in CD4^+^ T cell-B cell interactions and is one of the susceptibility genes for RA^2^,^29^. Five-day culture of PBMC with IL-21 increased the expression of CXCR4 on memory CD4^+^ T cells (Fig. 3A,B)^30^. Furthermore, addition of anti-HLA-DR antibody blocked this increase, suggesting that HLA-DR is directly involved in the elevation of CXCR4 expression (Fig. 3B). Meanwhile, strong TCR signaling induced by PMA decreased CXCR4 expression, consistent with reports that strong TCR stimulation leads to CXCR4 internalization^31^.

To elucidate the linkage between antigen-presenting cells and SE + RA memory CD4^+^ T cells with increased CXCR4 expression, we examined the association between CXCR4 expression on memory CD4^+^ T cells and HLA-DR expression on antigen-presenting cells. In accordance with the observation that B cells from SE + RA patients had significantly higher HLA-DR expression (Fig. 1C), there was a significant correlation between CXCR4 expression on memory CD4^+^ T cells and the amount of HLA-DR expressed on B cells (Fig. 3C), while HLA-DR expressed on monocytes and dendritic cells was not significantly correlated. This correlation between CXCR4 expression on memory CD4^+^ T cells and the amount of HLA-DR expressed on B cells may be specific to RA because it was not observed in HD (Figure S9). These results suggest that the increased CXCR4 expression on memory CD4^+^ T cells in RA patients is regulated both by inflammatory cytokines and HLA-DR expression on B cells.

### Transcriptome analysis of CXCR4-expressing memory CD4^+^ T cells

**Figure 4:**
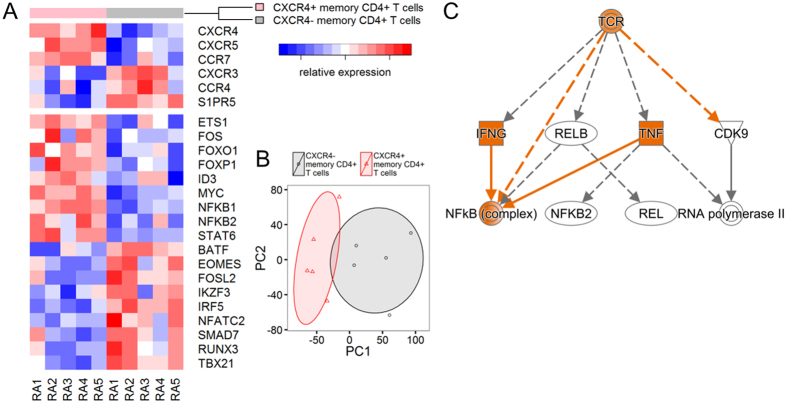
Transcriptome differences in CXCR4^+^ memory CD4^+^ T cells and CXCR4^−^ memory CD4^+^ T cells from RA patients. (**A**) Heatmap of selected receptors and transcription factors differentially expressed (FDR < 0.05) in CXCR4^+^ and CXCR4- memory CD4^+^ T cells from five RA patients with active disease (RA1-5). Red color represents the upregulation and blue color represents the downregulation of respective genes. (n = 5). (**B**) Principal component analysis of whole transcriptome to summarize differences between CXCR4^+^ and CXCR4^−^ memory CD4^+^ T cells. PC1 explained 24% and PC2 explained 12% of the total variance. (n = 5). (**C**) The IPA mechanistic network identified the TCR as the most significant upstream regulator of CXCR4^+^ memory CD4^+^ T cells upregulated genes (p = 1.67E-08, Z-score = 1.256). Orange color represents more confident predicted activation.

We compared the transcriptome of CXCR4^+^ memory CD4^+^ T cells of RA patients with CXCR4^−^ memory CD4^+^ T cells by RNA-seq. Chemokine receptors, such as CXCR4, CXCR5, and CCR7, and transcription factors, such as MYC, NFKB1, and NFKB2, were differentially upregulated in the CXCR4^+^ population (Fig. 4A). Comparison of the entire transcriptome of CXCR4^+^ and CXCR4^−^ memory CD4^+^ T cells revealed clear global differences (Fig. 4B), suggesting that CXCR4-expressing memory CD4^+^ T cells are a distinct subset in RA. Pathway analysis revealed that MYC-related protein synthesis and the mTOR pathway were activated in the CXCR4^+^ population (Figure S10), consistent with a previous report that showed that MYC controls metabolic reprogramming upon T cell activation^32^. Moreover, T cell receptor (TCR) signaling was the most significant upstream regulator of transcriptomic changes in the CXCR4^+^population (Fig. 4C and Figure S10D), in agreement with our observation that HLA-DR and CXCR4 expression on memory CD4^+^ T cells are associated (Fig. 3).

### CXCR4 expression predicts a better response to CTLA4-Ig in RA patients

**Figure 5:**
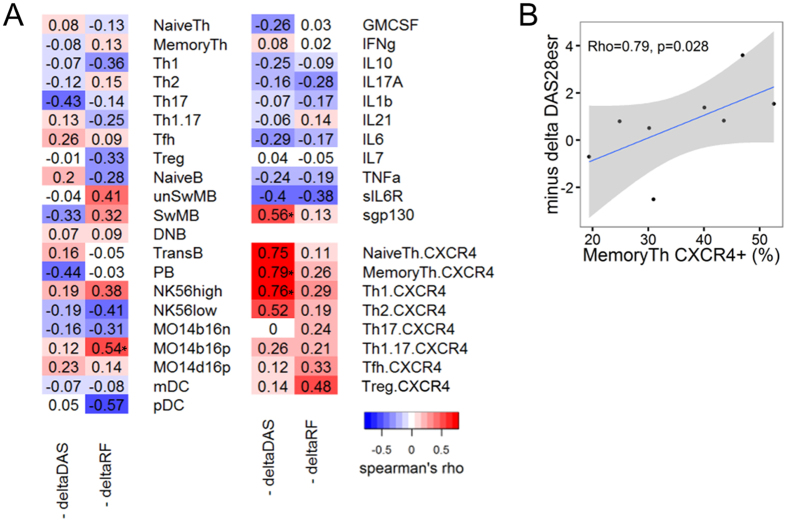
Increased CXCR4 expression on memory CD4^+^ T cells predicts a better response to CTLA4-Ig. (**A**) Correlations between baseline immunophenotyping subsets, cytokines, soluble cytokine receptors, and CXCR4 expression on CD4^+^ T cell subsets and clinical responses to CTLA4-Ig treatment in RA patients (n = 8–20). Tfh1, Tfh2, and Tfh17 subpopulations were eliminated from the analysis. (**B**) Representative scatter plot between baseline MemoryTh CXCR4 positivity (%) and DAS28esr change from baseline following CTLA4-Ig treatment (Minus delta DAS28esr; n = 8). *p < 0.05 Spearman’s test.

We next investigated the relationship between the therapeutic efficacy of biologics and immunological parameters examined above. Immunophenotyping and serum cytokine analysis were performed when CTLA4-Ig treatment was initiated and after 6 months of treatment in 20 RA patients. We also examined the clinical efficacy of CTLA4-Ig in RA patients, and found that it partially reverses immunological abnormalities in RA, specifically that CTLA4-Ig decreased the ratio of inflammatory CD14^bright^CD16^+^ monocytes and increased the ratio of mDCs (Figures S2 and S11)^33^,^34^,^35^. Decreased HLA-DR expression on T cells and decreased inflammatory cytokines were observed in parallel with a decrease in DAS28esr. CTLA4-Ig administration significantly reduced CXCR4 expression on naive CD4^+^ T cells (Figure S11D). Importantly, although CTLA4-Ig treatment did not decrease HLA-DR expression on B cells or CXCR4 expression on memory CD4^+^ T cells, higher CXCR4 expression on memory CD4^+^ T cells was predictive of a better response to CTLA4-Ig treatment (Fig. 5 and Figure S11). This result also confirms the importance of CXCR4 expression on memory CD4^+^ T cells in RA and supports its role as an immunological biomarker.

## Discussion

In spite of the importance of the HLA-DRB1 genotype for the development of ACPA-positive RA and bone destruction^5^,^7^, little is known about its association with lymphocyte subsets. In this study, we identified that the HLA-DRB1 disease-susceptible haplotype, SE, is related to PBMC memory CD4^+^ T cell subset and CXCR4 expression on memory CD4^+^ T cells (Figs 1B and 2B). Increased CXCR4 expression on memory CD4^+^ T cells from SE + RA patients may explain the linkage between HLA-DRB1 genotype and destructive arthritis (Fig. 2C and Table 1). The moderate correlation between CXCR4 expression on memory CD4^+^ T cells and DAS28esr/ HAQ suggests that synovial migration or retention of memory CD4^+^ T cells by increased CXCR4 expression is associated with sustained autoimmunity and local inflammation that eventually lead to functional disability due to bone destruction. Furthermore, the quantitative increase of HLA-DR expression on SE + RA B cells and its correlation to CXCR4 expression levels on memory CD4^+^ T cells suggest the importance of B cells as antigen-presenting cells in RA (Figs 1C and 3C). In addition, there were no significant differences between SE-HD and SE + HD in HLA-DR expression on B cells or CXCR4 expression on memory CD4^+^ T cells (Figures S3 and S5), which suggests that the relationship between SE and enhanced HLA-DR expression on B cells or enhanced CXCR4 expression on memory CD4^+^ T cells could be specific to RA. It has been reported that increased HLA-DR molecules on antigen-presenting cells, especially B cells in RA, can efficiently present low-affinity peptides to T cells^24^. Some factors other than HLA-DRB1 SE alleles may contribute to the increased HLA-DR expression on SE + RA B cells. Although stimulation related response expression quantitative trait locus (reQTL) effect^36^ of HLA-DRB1 SE alleles is one candidate mechanism under inflammation in RA, further examination is required to address this important point. Since TCR signaling is the most significant upstream regulator of transcriptomic changes in CXCR4-expressing memory CD4^+^ T cells (Fig. 4C), the quantitative increase in HLA-DR expression on B cells could contribute to the increased CXCR4 expression observed on memory CD4^+^ T cells in RA, and the increased amount of serum IL-21 might also play a role (Fig. 3B and Figure S4B). Furthermore, CXCR4 expression on the most expanded memory CD4^+^ T cell clones was up-regulated compared to non-expanded clones in the RA synovium^37^. However, it is possible that increased CXCR4 expression on memory CD4^+^ T cells reflects non-antigen-specific T cell activation because increased CXCR4 expression was observed in various CD4^+^ T cell subsets, and strong TCR signaling decreased CXCR4 expression *in vitro* (Fig. 3B). Inflammatory cytokines, especially TNF-α and IL-6, can also activate CD4^+^ T cells and contribute to increased CXCR4 expression^21^. Regardless, inflammatory cytokine concentrations were not associated with HLA-DRB1 genotype or RA disease activity (Figure S4). Our data strongly suggest the close link between HLA-DR, CXCR4-expressing memory CD4^+^ T cells, and disease activity in RA.

In spite of the higher expression of CXCR4 on naive CD4^+^ T cells compared to memory CD4^+^ T cells, CXCR4 expression on naive CD4^+^ T cells was not associated with RA disease activity (Fig. 2 and Table S3). Although CXCR4 is a receptor for HIV-1 entry into CD4^+^ cells, memory CD4^+^ T cells are preferentially infected and harbor more integrated proviral DNA than naive CD4^+^ T cells^38^. It has been reported that memory CD4^+^ T cells possess higher cortical actin and chemotactic actin activity than naive CD4^+^ T cells, thus showing higher susceptibility to CXCL12 and HIV-1^39^. This implies that there is a functional difference between CXCR4 on memory CD4^+^ T cells and CXCR4 on naive CD4^+^ T cells, which may be related to the preferential association between CXCR4-expressing memory CD4^+^ T cells and RA disease activity.

ACPA had a stronger correlation to HLA-DRB1 SE and clinical resistance to therapy than RF^40^. Our data suggest different mechanisms for the generation of these two autoantibodies. We showed that RF is significantly correlated with the frequency of plasmablasts and inflammatory cytokines (Fig. 1A and Figure S4A), which suggests that the inflammatory environment and B cell activation may induce the production of RF. Meanwhile, ACPA was correlated with the frequency of memory CD4^+^ T cells, suggesting a role for acquired immunity to citrullinated peptides in the pathogenesis of ACPA, especially in SE + RA patients.

This study identified a previously unrecognized predictor for the efficacy of CTLA4-Ig. Increased CXCR4 expression on memory CD4^+^ T cells predicted a better response to CTLA4-Ig (Fig. 5). We did not observe any direct effects of CTLA4-Ig on CXCR4 expression on memory CD4^+^ T cells or HLA-DR expression on B cells (Figure S11). However, because CTLA4-Ig significantly suppressed CXCR4 expression on naive CD4^+^ T cells (Figure S11D), it is possible that CTLA4-Ig may suppress CXCR4 expression on memory CD4^+^ T cells after a longer period of treatment. Clinically, a better response to CTLA4-Ig has been reported for RA patients with high ACPA titers^41^. CTLA4-Ig blocks CD28 costimulation, thereby interfering with the interaction between CD4^+^ T cells and antigen-presenting cells, such as B cells. Increased CXCR4 expression on memory CD4^+^ T cells, which also corresponds to high ACPA titers (Figure S6), could be related to stronger interactions between CD4^+^ T cells and B cells. Another possibility is that higher CXCR4 expression may be associated with enhanced migration of memory CD4^+^ T cells to the joints. Monocytes/macrophages could produce inflammatory cytokines upon interactions with the migrated CD4^+^ T cells, and this pathogenic process may be inhibited by CTLA4-Ig. Further research is needed to elucidate the mechanism of the improved CTLA4-Ig responses in patients with higher CXCR4 expression.

In summary, we revealed genetic-immunological interactions that impact RA pathogenesis. Our data show that increased CXCR4 expression on memory CD4^+^ T cells correlates with disease activity and response to CTLA4-Ig treatment. Therefore, an immunological abnormality that induces enhanced HLA-DR expression on B cells and CXCR4 expression on RA memory CD4^+^ T cells may impact RA pathology and could potentially be a therapeutic target.

## Methods

### RA patients and healthy donors

We recruited 91 RA patients from April 2013 to March 2015 who fulfilled the 1987 revised American College of Rheumatology Criteria or the 2010 American College of Rheumatology/European League Against Rheumatism classification criteria. Patients with active infection or malignancy were excluded. The following clinical data were collected (Table S1): age, sex, disease duration, methotrexate (MTX) usage, conventional and biological disease-modifying antirheumatic drugs (DMARDs) usage, rheumatoid factor (RF) titer, anti-cyclic citrullinated peptide antibody (ACPA) titer, Disease Activity Score 28 joints-ESR (DAS28esr) and Health Assessment Questionnaire Disability Index (HAQ)^10^,^11^. RF titers were measured with latex coagulating nephelometry (cut off value is 15 IU/ml). ACPA titers were measured by chemiluminescence enzyme immunoassay (cut of value is 4.5 U/ml, Medical and Biological Laboratories, Japan). 22 patients were recruited at the initiation of CTLA4-Ig treatment, and 20 patients that continued treatment were reanalyzed after 6 months. Two patients discontinued CTLA4-Ig treatment due to ineffectiveness and pneumonia. Synovial fluid samples were derived from therapeutic arthrocentesis. 110 healthy donors (HD) were screened with a questionnaire to disqualify HD with diseases requiring treatment. All donors provided written informed consent, and the use of human peripheral blood and synovial fluid samples was approved by the Ethical Committee of the University of Tokyo Hospital (No. 10154 and G3582). The methods were carried out in accordance with the approved guidelines.

### Human PBMC isolation and flow cytometry

Preliminary immunophenotyping analyses with standard cryopreserved samples exhibited substantial differences from fresh samples^42^. Therefore, all collected samples were freshly analyzed by flow cytometry. Human peripheral blood mononuclear cells (PBMC) were isolated by Ficoll-Paque Plus density gradient centrifugation (GE healthcare). Erythrocytes were lysed with ammonium chloride potassium buffer, and non-specific binding was blocked with Fc-gamma receptor antibodies. Staining was performed with predefined cell and antibody concentrations. The following antibodies were used: Human Fc Receptor Binding Inhibitor Purified (eBioscience), CD3-PE-Cy7 (UCHT1, BioLegend), CD3-PerCP-Cy5.5 (UCHT1, BioLegend), CD4-PerCP-Cy5.5 (OKT4, BioLegend), CD4-V500 (RPA-T4, BD Biosciences), CD11c-Brilliant Violet 421 (B-ly6, BD Biosciences), CD14-FITC (M5E2, BioLegend), CD16-PerCP-Cy5.5 (3G8, BioLegend), CD19-APC-Cy7 (HIB19, BioLegend), CD19-V500 (HIB19, BD Biosciences), CD24-PE (ML5, BD Biosciences), CD25-Brilliant Violet 421 (BC96, BioLegend), CD25-PE-Cy7 (BC96, eBioscience), CD27-FITC (O323, eBioscience), CD38-PE-Cy7 (HIT2, BioLegend), CD45RA-APC-Cy7 (HI100, BioLegend), CD56 -APC-Cy7 (HCD56, BioLegend), CD123-APC (AC145, Miltenyi), CD127-PE-Cy7 (eBioRDR5, eBioscience), CXCR3-Brilliant Violet 421 (1C6, BD Biosciences), CXCR4-APC (12G5, BD Biosciences), CXCR5-Alexa Fluor 488 (RF8B2, BD Biosciences), CCR6-PE (11A9, BD Biosciences), CCR7-PerCP-Cy5.5 (G043H7, Biolegend), HLA-DR-PE (L243, eBioscience), IgD-Brilliant Violet 421 (IA6-2, BD Biosciences). Flow cytometric analysis was performed on an 8-color MoFlo XDP (Beckman Coulter).

### Immunophenotyping

Subset definitions are summarized in Table S2. We classified CD4^+^ T cells, B cells, natural killer (NK) cells, monocytes, and dendritic cells (DC) based on Human Immunology Project classification^9^ and also added modifications for subsets already reported to be important in RA. First, for the classification of CD4^+^ T cells, the follicular helper T cell (Tfh) subset and its subclassifications were added because Tfh, a specialized CD4^+^ T cell subset that helps B cell differentiation and antibody production, could potentially be pathogenic in RA (Figure S1)^43^,^44^,^45^,^46^. Second, for the classification of monocytes, HLA-DR positivity was added to rigorously exclude granulocytes, and three precise subset classifications were employed based on previous reports showing the expansion and high inflammatory cytokine production of CD14^bright^CD16^+^ monocytes in RA^33^,^47^,^48^. The ratio of each subset is represented as the percentage of the parent population (Table S2). CXCR4-positive ratios were determined based on isotype controls (APC Mouse IgG2a Kappa Isotype Control, MOPC-173, Biolegend). As CXCR4-positive ratios on CD4^+^ T cells were correlated with age^49^, we adjusted CXCR4-positive ratios of each CD4^+^ T cell subset by linear regression for age when comparing HD and RA.

### HLA-DRB1 typing and quantitative HLA-DR expression analysis

The HLA-DRB1 alleles of RA patients were genotyped by the polymerase chain reaction-sequence-based typing (PCR-SBT) method (SRL, Japan). HD were genotyped by Infinium OmniExpressExome (Illumina). Quality control (QC) of genotyping data was performed by PLINK 1.90^50^. PCA of genotype data was performed by EIGENSTRAT^51^. Pre-phasing was performed by SHAPEIT and imputation was performed by IMPUTE2 with 1000 Genome Project Phase1 version 3 reference data^52^,^53^. Four-digit HLA alleles were imputated with SNP2HLA software^54^,^55^. The following alleles were considered to carry shared epitopes; 01:01, 01:02, 04:01, 04:04, 04:05, 04:08, 04:10, 10:01, 14:02, 14:06^56^. Using a monoclonal antibody to a common epitope of HLA-DR, cell surface HLA-DR quantitation analysis was performed with QuantiBrite PE (BD Biosciences), as instructed. The HLA-DR-PE antibody concentration we used was confirmed to saturate cell-surface HLA-DR^24^.

### Multiplex cytokine analysis

Serum samples were stored at −30 °C until assays were performed. To reduce false amplification by heterophilic antibodies, HeteroBlock (Omega Biologicals) was added to all serum samples to achieve a final concentration of 150 μg/ml^57^. Serum concentrations of GM-CSF, IFN-γ, IL-10, IL-17A, IL-1b, IL-21, IL-4, IL-6, TNF-α, IL-7, sIL-6R and spg130 were measured using the Milliplex MAP kit (the Human Soluble Cytokine Receptor Magnetic Bead Panel and the Human High Sensitivity T Cell Magnetic Panel, Merck Millipore) and the BioPlex 3D system (Bio-Rad), according to the manufacturer’s instructions. To avoid inter-assay variation, all samples were analyzed at one time with the same kit lot number. Because IL-4 concentrations were above the detection limit only in 3.8% (3/78) of the samples, IL-4 was excluded from further analysis. Other cytokines or soluble cytokine receptors were detected in 88–100% of the samples.

### PBMC culture

PBMC samples (1 × 10^6^ cells/mL) were cultured in RPMI 1640 medium supplemented with 10% FBS (Equitech-Bio), 2 mM γ-glutamine (Sigma), 100 U/ml penicillin (Sigma), and 100 μg/ml Streptomycin (Sigma) in a humidified incubator at 37 °C, 5% CO2, for 120 hours. Recombinant human IL-21 (Peprotech) was added at 10 ng/ml. Phorbol myristate acetate (PMA, Sigma) was added at 25 ng/ml. To block the association between HLA-DR and T cell receptors, anti-HLA DR monoclonal antibodies (L243, Biolegend) were added to final concentrations of 0.1 μg/mL, 1 μg/mL, and 10 μg/mL. Viable 7-Amino-Actinomycin D (7-AAD, Biolegend)-negative CD3^+^ CD4^+^ CD45RA- cells were analyzed for CXCR4 expression with flow cytometry.

### RNA-seq

CD3^+^CD4^+^CD45RA^-^CXCR4^+^ cells (CXCR4^+^ memory CD4^+^ T cells) and CD3^+^CD4^+^CD45RA^−^CXCR4^−^ cells (CXCR4^-^ memory CD4^+^ T cells) were sorted from five RA patients with active disease (DAS28esr > 3.2) by MofloXDP. Upper and lower 33rd percentiles of CXCR4 expression were considered to be CXCR4^+^ and CXCR4^-^ populations of memory CD4^+^ T cells, respectively, in this RNA-seq analysis. 4.0–10 × 10^4^ cells were sorted and stored at −80 ^o^C until use. Library preparation was performed using a TruSeq Stranded mRNA Sample Prep Kit (Illumina). For PCR enrichment of the library, an additional 20 cycles were added to the manufacturer’s instructions because of the limited RNA input. The quality of the libraries was validated on an Agilent Technologies 2100 Bioanalyzer, and paired-end sequencing was performed by MiSeq (Illumina). 1.0–2.1 × 10^6^ reads were obtained for each sample. Illumina Truseq adaptors were trimmed by Cutadapt^58^ and low-quality ends (phred score < 20) were also trimmed by FASTX-Toolkit (http://hannonlab.cshl.edu/fastx-toolkit). Mapping was performed with STAR^59^ with UCSC hg19 reference sequence (http://genome.ucsc.edu/). The uniquely mapped rate was 93.08 to 94.63%. Assignment of reads to genes was performed by HTSeq^60^ based on the UCSC gene model. Differential expression analysis was performed with a generalized linear model based edgeR^61^. 922 genes were upregulated in CXCR4^high^ memory CD4^+^ T cells and 997 genes were downregulated with FDR < 0.05. Log_2_ counts-per-million (logCPM) values for each gene were used for heatmap and principal component analysis. Network analysis of upregulated and downregulated genes was performed with Ingenuity Pathways Analysis (IPA, Ingenuity).

### Statistics

Categorical data were tested with Fisher’s exact test. Normality was tested with the Kolmogorov–Smirnov test. Differences between groups of normally distributed continuous data were tested with unpaired t-tests with Bonferroni corrections. Differences between groups of non-normally distributed continuous data were tested for significance as follows: nonparametric Mann–Whitney U test to compare two groups and the Kruskal–Wallis test with post-hoc Wilcoxon test with Bonferroni corrections to compare three groups. Correlations were evaluated by nonparametric Spearman’s rank correlation coefficients. Missing values were excluded pairwise in the correlation analysis. Samples with missing values were excluded from principal component analysis. CD4^+^ T cell subsets were examined for an association between CXCR4 expression and DAS28esr by univariate and multivariate linear regression analyses. Age, sex, disease duration, shared epitope positivity, RF titer, and ACPA titer were added as covariates. In the multivariate regression model selection, an exhaustive search was performed to maximize adjusted R-squared. Least absolute shrinkage and selection operator (LASSO) regression was also performed^62^. P values less than 0.05 were considered significant. All statistical analyses were performed with R version 3.1.2 (R Foundation for Statistical Computing).

## Additional Information

**How to cite this article**: Nagafuchi, Y. *et al*. Immunophenotyping of rheumatoid arthritis reveals a linkage between HLA-DRB1 genotype, CXCR4 expression on memory CD4^+^ T cells, and disease activity. *Sci. Rep.* **6**, 29338; doi: 10.1038/srep29338 (2016).

## Supplementary Material

Supplementary Information

## Figures and Tables

**Table 1:** Univariate and multivariate relationships between CD4^+^ T cell subsets and DAS28esr.

variable	Univariate regression		Multivariate regression	
	β	P value	β	P value
age	0.19	0.08	Not selected	Not selected
sex	−0.033	0.76	Not selected	Not selected
disease duration	−0.0087	0.94	−0.24	0.14
Shared epitope positivity	0.028	0.79	Not selected	Not selected
RF titer	0.11	0.32	Not selected	Not selected
ACPA titer	0.25	0.022*	0.17	0.32
Th1	0.08	0.47	Not selected	Not selected
Th2	0.2	0.07	Not selected	Not selected
Th17	−0.0072	0.94	−0.24	0.15
Tfh	0.061	0.58	Not selected	Not selected
Treg	0.043	0.71	Not selected	Not selected
MemoryTh.CXCR4	0.52	0.0018**	0.54	0.0023**

RA n = 32. *p < 0.05, **p < 0.01.

RF, Rheumatoid Factor; ACPA, anti-cyclic citrullinated peptide antibody; DAS28esr, Disease Activity Score 28 joints-ESR.
