# Supplementary material for: Immunophenotyping of rheumatoid arthritis reveals a linkage between HLA-DRB1 genotype, CXCR4 expression on memory CD4+ T cells, and disease activity
Source: Sci Rep. 2016 Jul 7;6:29338. doi: 10.1038/srep29338 (PMC4935954; doi:10.1038/srep29338)
Supplement: Supplementary Information [file srep29338-s1.pdf]

Supplementary Material

Title:

Immunophenotyping of rheumatoid arthritis reveals a linkage between HLA-DRB1 genotype, CXCR4 expression on memory $CD4^+$ T cells, and disease activity.

Authors:

Yasuo Nagafuchi[1], Hirofumi Shoda[1], Shuji Sumitomo[1], Shinichiro Nakachi[1], Rika Kato[1], Yumi Tsuchida[1], Haruka Tsuchiya[1], Keiichi Sakurai[1], Norio Hanata[1], Shoko Tateishi[1], Hiroko Kanda[1], Kazuyoshi Ishigaki[1, 2], Yukinori Okada[3], Akari Suzuki[2], Yuta Kochi[2], Keishi Fujio[1, *], Kazuhiko Yamamoto[1, 2]

Affiliations:

[1]Department of Allergy and Rheumatology, Graduate School of Medicine, The University of Tokyo, Tokyo, Japan.

[2]Laboratory for Autoimmune Diseases, Center for Integrative Medical Sciences, RIKEN, Yokohama, Japan.

[3]Department of Statistical Genetics, Osaka University Graduate School of Medicine, Osaka, Japan.

*To whom correspondence should be addressed.

E-mail: kfujio-tky@umin.ac.jp

Supplementary Figure Legends

Figure S1. Immunophenotyping by flow cytometry.

Peripheral blood mononuclear cells (PBMC) $CD3^{+}CD4^{+}$ helper T cell subsets were classified based on CD45RA, CXCR3 (Th1 marker), CCR6 (Th17 marker), and CXCR5 (Tfh marker).

Figure S2. Immunophenotyping of RA patients.

Comparison of 24 immunophenotyped subset ratios among healthy donors (HD), shared epitope (SE)-negative RA patients, and SE-positive RA patients (HD; n=110, SE-RA; n=30, SE+RA; n=61).

*p<0.05 **p<0.01 ***P<0.001 Kruskal-Wallis tests with post-hoc Wilcoxon tests with Bonferroni corrections.

Figure S3. RA specific association between SE and B cell HLA-DR expression.

Comparison of HLA-DR expression on B cells between shared epitope (SE)-negative healthy donors (HD), SE-positive HD, SE-negative RA patients, and SE-positive RA patients (SE-HD; n=63, SE+HD; n=47, SE-RA; n=30, SE+RA; n=61). The analysis is based on flow cytometric HLA-DR quantitative expression per cells.

**p<0.01 Kruskal-Wallis tests with post-hoc Wilcoxon tests with Bonferroni corrections.

Figure S4. RF titer is related to dysregulated inflammatory cytokines.

(A) Correlations between 11 cytokines and soluble cytokine receptors and four clinical parameters in RA patients (n=30). Representative scatter plots are also shown on the right, as in figure 1A.

(B) Comparison of serum cytokine/soluble cytokine receptor concentrations among healthy donors (HD), shared epitope (SE)-negative RA patients, and SE-positive RA patients (HD; n=34, SE-RA; n=11, SE+RA; n=19).

(C) Principal component analysis of 11 cytokine and soluble cytokine receptors to summarize the difference between HD, SE-RA, and SE+RA. PC1 explained 48% and PC2 explained 13% of the total variance (HD; n=26, SE-RA; n=10, SE+RA; n=12).
*$p<0.05$ **$p<0.01$ ***$P<0.001$ Spearman's tests (A) or Kruskal-Wallis tests with post-hoc Wilcoxon tests with Bonferroni corrections (B).

Figure S5. RA specific association between SE and CXCR4.
Comparison of CXCR4 expression on CD4$^+$ T cell subsets among SE-negative HD, SE-positive HD, SE-negative RA patients, and SE-positive RA patients (SE-HD; n=46, SE+HD; n=36, SE-RA; n=8, SE+RA; n=27).
***$P<0.001$ Kruskal-Wallis tests with post-hoc Wilcoxon tests with Bonferroni corrections.

Figure S6. Enhanced CXCR4 expression on CD4$^+$ T cells of RA patients with high ACPA titers.
Comparison of CXCR4 expression on CD4$^+$ T cell subsets among healthy donors (HD), ACPA-negative RA patients (ACPA-), RA patients with low ACPA titers (ACPA+, 4.5 U/ml ≤ ACPA titer < 100 U/ml), and RA patients with high ACPA titers (ACPA++, 100 U/ml ≤ ACPA titer). HD; n=82, ACPA-; n=5 (SE-; n=3, SE+; n=2), ACPA+; n=11 (SE-; n=3, SE+; n=8), ACPA++; n=16 (SE-; n=2, SE+; n=14).
*$p<0.05$ **$p<0.01$ ***$P<0.001$ Kruskal-Wallis tests with post-hoc Wilcoxon tests with Bonferroni corrections.

Figure S7. CXCR4 expression on B cell subsets.
Comparison of CXCR4 expression on B cell subsets among healthy donors (HD), shared epitope (SE)-negative RA patients, and SE-positive RA patients (HD; n=82, SE-RA; n=8, SE+RA; n=27). ***$P<0.001$

Kruskal-Wallis tests with post-hoc Wilcoxon tests with Bonferroni corrections.

Figure S8. CCR7 expression on $CD4^+$ T cell subsets.
Comparison of $CD4^+$ T cell subsets classified according to CCR7 and CD45RA expression among healthy donors (HD), shared epitope (SE)-negative RA patients, and SE-positive RA patients (HD; n=108, SE-RA; n=25, SE+RA; n=56). $CD3^+CD4^+$ T cells were defined as naive $CD4^+$ T cells ($CCR7^+CD45RA^+$ Naive), central memory $CD4^+$ T cells ($CCR7^+CD45RA^-$ CM), effector memory $CD4^+$ T cells ($CCR7^-CD45RA^-$ EM), and effector $CD4^+$ T cells ($CCR7^-CD45RA^+$ Effector). No significant differences were observed between groups by Kruskal-Wallis tests with post-hoc Wilcoxon tests with Bonferroni corrections.

Figure S9. B cell HLA-DR levels and CXCR4 expression on memory $CD4^+$ T cells in HD.
Scatter plots between B cell, monocyte (MO), and dendritic cell (DC) HLA-DR quantitative expression per cells and CXCR4-positive ratios of the MemoryTh subset in HD (n=82) compared to RA patients (Figure 3C).

Figure S10. Pathway analysis of $CXCR4^+$ memory $CD4^+$ T cells.
(A) Top network of differentially expressed genes in $CXCR4^+$ and $CXCR4^-$ memory $CD4^+$ T cells by IPA pathway analysis. Red color represents upregulation and green color represents downregulation in $CXCR4^+$ $CD4^+$ T cells.
(B-C) Most significantly upregulated (B) and downregulated (C) pathways in $CXCR4^+$ memory $CD4^+$ T cells. Top 3 pathways in IPA canonical pathway analysis with -log(p-value) greater than 12.5 and an absolute z-score greater than 2 are shown.

(D) TCR downstream genes differentially upregulated in CXCR4$^+$ memory CD4$^+$ T cells. Genes were selected by IPA knowledge database.

Figure S11. CTLA4-Ig treatment partially restores immunological abnormalities in RA.
Effects of 6 months of CTLA4-Ig treatment on PBMC immunophenotyping (A; n=20), HLA-DR expression (B; n=20), serum cytokine and soluble cytokine receptor concentrations (C; n=14), CXCR4 expression on CD4$^+$ T cells (D; n=8), and clinical disease activity (E; n=20). Percent changes from baseline values are plotted.
*p<0.05 **p<0.01 ***P<0.001 Paired Wilcoxon test.

Table S1. Baseline characteristics

	Shared epitope negative RA (SE-)	Shared epitope positive RA (SE+)	p-values	Healthy Donors (HD)
n	30	61		110
age, median (IQR)	56 (46-64)	64 (52-67)	0.18	39 (31-45)
sex (f/m)	26/4	47/14	0.4	88/22
disease duration, years, median (IQR)	3 (1-14)	6 (1-13)	0.34	
RF, IU/ml, median (IQR)	43 (21-182)	72 (31-200)	0.20	
ACPA, U/ml, median (IQR)	40 (1.8-94)	102 (31-348)	0.0030 **	
DAS28esr, median (IQR)	4.3 (3.4-5.3)	4.5 (3.7-5.3)	0.79	
HAQ, median (IQR)	0.63 (0.13-1.2)	0.88 (0.25-1.9)	0.29	
treatment naïve, n (%)	7 (23)	10 (17)	0.57	
treatment with conventional DMARDs, n (%)	24 (77)	50 (83)	0.57	
treatment with methotrexate, n (%)	12 (39)	30 (50)	0.0058 **	
treatment with biologic DMARDs, n (%)	7 (23)	14 (23)	1	

Categorical data were tested with Fisher's exact test. Continuous data were tested with Mann-Whitney U test. **p<0.01

RF, Rheumatoid Factor; ACPA, anti-cyclic citrullinated peptide antibody; DAS28esr, Disease Activity Score 28 joints-ESR; HAQ, Health Assessment Questionnaire Disability Index; DMARDs, disease-modifying antirheumatic drugs

Table S2. Immunophenotyping subset definitions

subset name (abbreviations)	cell definition
$CD4^+$ T cells	CD3+CD4+ (parent population)
naive $CD4^+$ T cells (NaiveTh)	CD3+CD4+CD45RA+
memory $CD4^+$ T cells (MemoryTh)	CD3+CD4+CD45RA-
T helper type 1cells (Th1)	CD3+CD4+CD45RA-CXCR5-CXCR3+CCR6-
T helper type 2 cells (Th2)	CD3+CD4+CD45RA-CXCR5-CXCR3-CCR6-
T helper type 17 cells (Th17)	CD3+CD4+CD45RA-CXCR5-CXCR3-CCR6+
T helper type1/17 cells (Th1.17)	CD3+CD4+CD45RA-CXCR5-CXCR3+CCR6+
follicular helper T cells (Tfh)	CD3+CD4+CD45RA-CXCR5+
follicular helper T cells/Th1 cells (Tfh1)	CD3+CD4+CD45RA-CXCR5+CXCR3+CCR6-
follicular helper T cells/Th2 cells (Tfh2)	CD3+CD4+CD45RA-CXCR5+CXCR3-CCR6-
follicular helper T cells/Th17 cells (Tfh17)	CD3+CD4+CD45RA-CXCR5+CXCR3-CCR6+
regulatory T cells (Treg)	CD3+CD4+CD45RA-CD25+CD127low
B cells	CD3-CD19+ (parent population)
naive B cells (NaiveB)	CD3-CD19+IgD+CD27-
unswitched memory B cells (unSwMB)	CD3-CD19+IgD+CD27+
switched memory B cells (SwMB)	CD3-CD19+IgD-CD27+
Double-negative B cells (DNB)	CD3-CD19+IgD-CD27-
transitional B cells (TransB)	CD3-CD19+CD24highCD38high
plasmablasts (PB)	CD3-CD19+IgD-CD27highCD38high
natural killer cells (NK)	CD3-CD19-CD14-CD56+ (parent population)
$CD56^{high}$ NK cells (NK56high)	CD3-CD19-CD14-CD56highCD16-
$CD56^{low}$ NK cells (NK56low)	CD3-CD19-CD14-CD56lowCD16+
monocytes	CD3-CD19-CD56-HLA-DR+CD14+ (parent population)
$CD14^{bright}CD16^-$ monocytes (MO14b16n)	CD3-CD19-CD56-HLA-DR+CD14brightCD16-
$CD14^{bright}CD16^+$ monocytes (MO14b16p)	CD3-CD19-CD56-HLA-DR+CD14brightCD16+
$CD14^{dim}CD16^+$ monocytes (MO14d16p)	CD3-CD19-CD56-HLA-DR+CD14dimCD16+
dendritic cells	CD3-CD19-CD56-HLA-DR+CD14- (parent population)
myeloid dendritic cells (mDC)	CD3-CD19-CD56-HLA-DR+CD14-CD11c+CD123-
plasmacytoid dendritic cells (pDC)	CD3-CD19-CD56-HLA-DR+CD14-CD11c-CD123+

Table S3. Univariate and multivariate relationships between CXCR4 expression on $CD4^+$ T cell subsets and DAS28esr

variable	Univariate regression		Multivariate regression	
	ß	P value	ß	P value
age	0.19	0.08	Not selected	
sex	-0.033	0.76	Not selected	
disease duration	-0.0087	0.94	-0.18	0.26
shared epitope positivity	0.028	0.79	Not selected	
RF titer	0.11	0.32	Not selected	
ACPA titer	0.25	0.022*	Not selected	
NaiveTh.CXCR4	0.35	0.043*	Not selected	
MemoryTh.CXCR4	0.52	0.0018**	0.52	0.0023**
Th1.CXCR4	0.46	0.0059**	Not selected	
Th2.CXCR4	0.42	0.013*	Not selected	
Th17.CXCR4	0.41	0.016*	Not selected	
Th1.17.CXCR4	0.45	0.0078**	Not selected	
Tfh.CXCR4	0.45	0.0074**	Not selected	
Tfh1.CXCR4	0.45	0.0080**	Not selected	
Tfh2.CXCR4	0.45	0.0070**	Not selected	
Tfh17.CXCR4	0.45	0.0074**	Not selected	
Treg.CXCR4	0.43	0.012*	Not selected	

RA n=32. *p<0.05, **p<0.01

RF, Rheumatoid Factor; ACPA, anti-cyclic citrullinated peptide antibody; DAS28esr, Disease Activity Score 28 joints-ESR

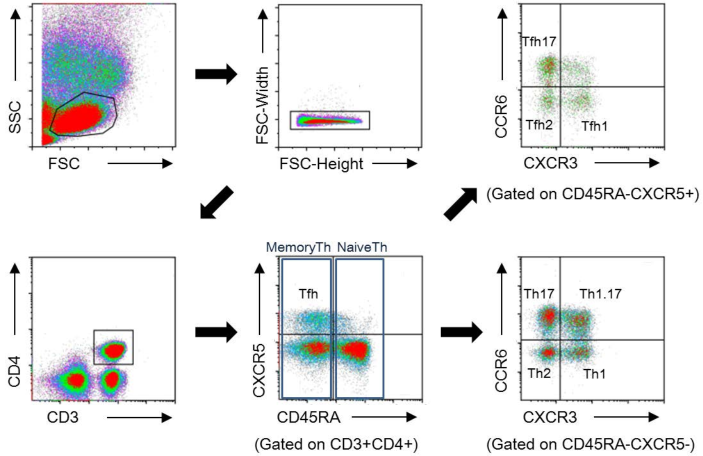
SSC
FSC
FSC-Width
FSC-Height
CCR6
CXCR3
Tfh17
Tfh2
Tfh1
(Gated on CD45RA-CXCR5+)
CD4
CD3
MemoryTh
NaiveTh
Tfh
CXCR5
CD45RA
(Gated on CD3+CD4+)
Th17
Th1.17
Th2
Th1
CCR6
CXCR3
(Gated on CD45RA-CXCR5-)

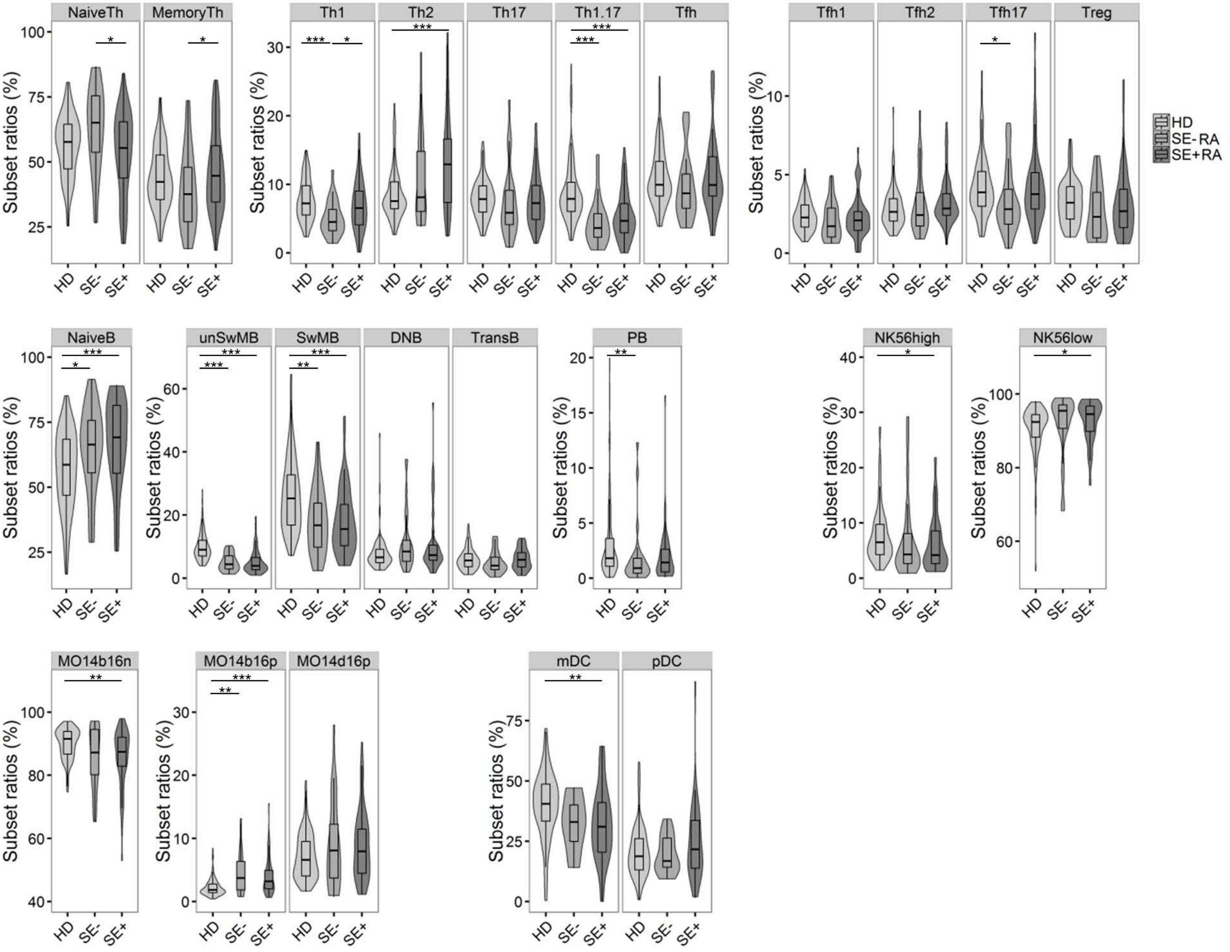

NaiveTh
MemoryTh
Th1
Th2
Th17
Th1.17
Tfh
Tfh1
Tfh2
Tfh17
Treg
HD
SE-RA
SE+RA
NaiveB
unSwMB
SwMB
DNB
TransB
PB
NK56high
NK56low
MO14b16n
MO14b16p
MO14d16p
mDC
pDC
Subset ratios (%)
HD
SE-
SE+

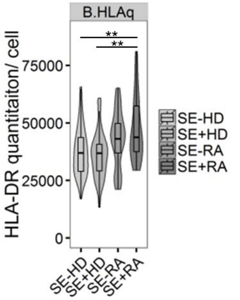

B.HLAq
HLA-DR quantitaiton/ cell
**
**
75000
50000
25000
0
SE-HD
SE+HD
SE-RA
SE+RA
SE-HD
SE+HD
SE-RA
SE+RA

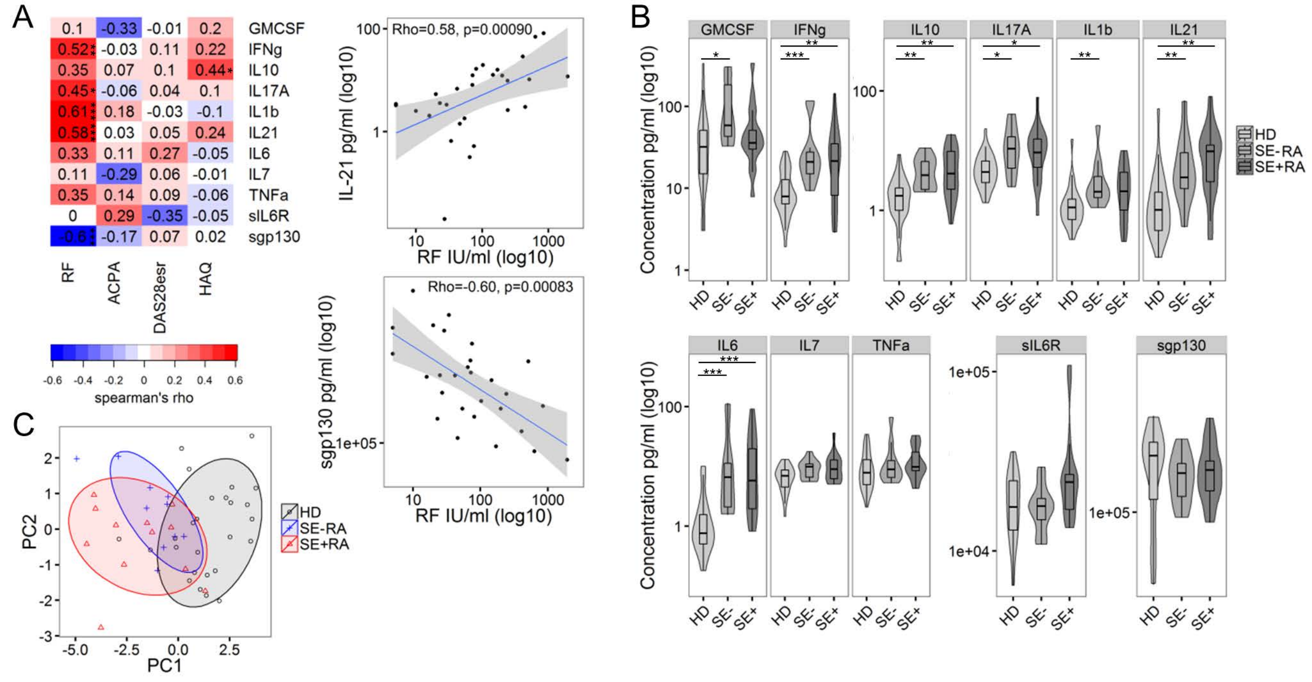

A
B
C
GMCSF
IFNg
IL10
IL17A
IL1b
IL21
IL6
IL7
TNFa
sIL6R
sgp130
RF
ACPA
DAS28esr
HAQ
spearman's rho
Rho=0.58, p=0.00090
IL-21 pg/ml (log10)
RF IU/ml (log10)
Rho=-0.60, p=0.00083
sgp130 pg/ml (log10)
Concentration pg/ml (log10)
HD
SE-RA
SE+RA
PC1
PC2

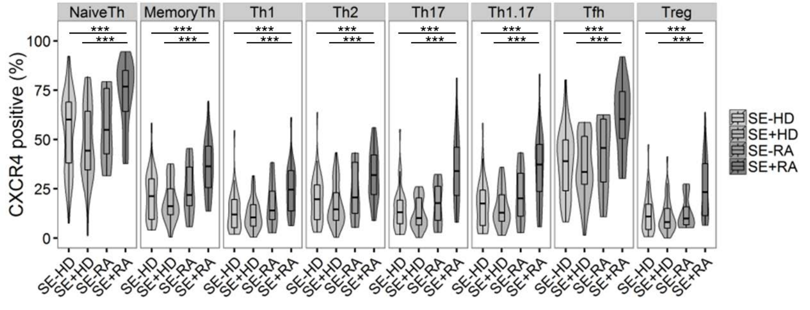
NaiveTh
MemoryTh
Th1
Th2
Th17
Th1.17
Tfh
Treg
***
***
CXCR4 positive (%)
100
75
50
25
0
SE-HD
SE+HD
SE-RA
SE+RA

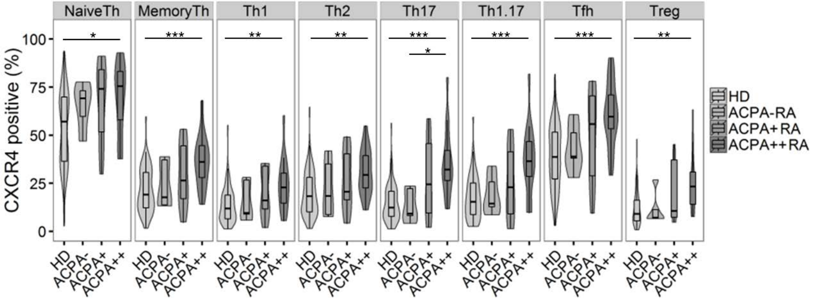
NaiveTh
MemoryTh
Th1
Th2
Th17
Th1.17
Tfh
Treg
CXCR4 positive (%)
100
75
50
25
0
HD
ACPA-
ACPA+
ACPA++
HD
ACPA-RA
ACPA+RA
ACPA++RA
*
***
**
**
***
*
***
***
**

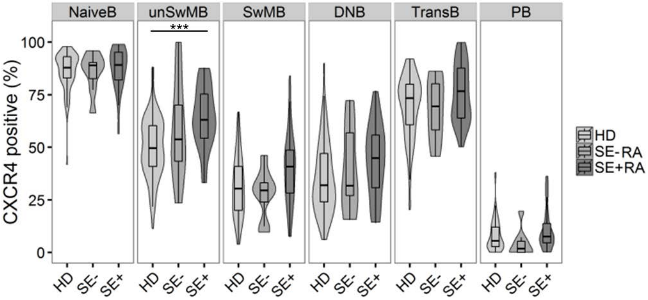

NaiveB
unSwMB
SwMB
DNB
TransB
PB
***
CXCR4 positive (%)
100
75
50
25
0
HD
SE-
SE+
HD
SE-RA
SE+RA

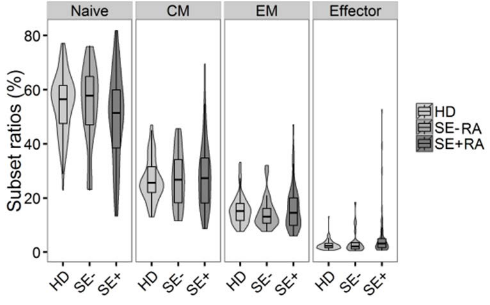

Naive
CM
EM
Effector
Subset ratios (%)
80
60
40
20
0
HD
SE-
SE+
HD
SE-RA
SE+RA

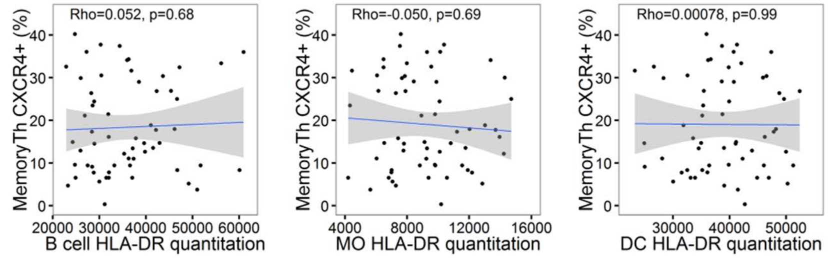

Rho=0.052, p=0.68
MemoryTh CXCR4+ (%)
0
10
20
30
40
20000
30000
40000
50000
60000
B cell HLA-DR quantitation
Rho=-0.050, p=0.69
MemoryTh CXCR4+ (%)
0
10
20
30
40
4000
8000
12000
16000
MO HLA-DR quantitation
Rho=0.00078, p=0.99
MemoryTh CXCR4+ (%)
0
10
20
30
40
30000
40000
50000
DC HLA-DR quantitation

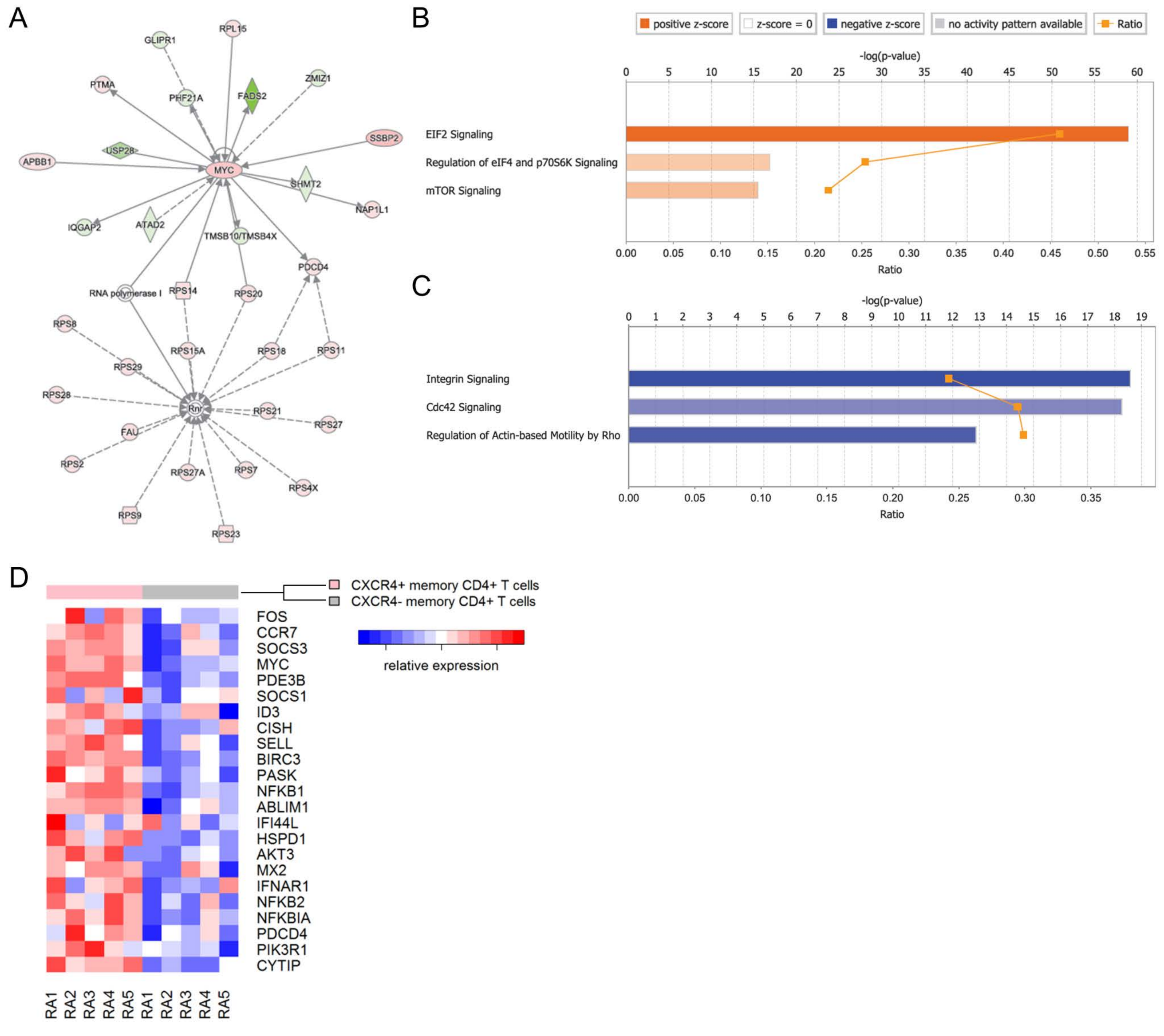
A
GLIPR1
RPL15
PTMA
PHF21A
FADS2
ZMIZ1
SSBP2
USP28
APBB1
MYC
SHMT2
NAP1L1
IQGAP2
ATAD2
TMSB10/TMSB4X
PDCD4
RNA polymerase I
RPS14
RPS20
RPS8
RPS15A
RPS18
RPS11
RPS29
RPS28
Rnr
RPS21
RPS27
FAU
RPS2
RPS27A
RPS7
RPS4X
RPS9
RPS23
B
positive z-score
z-score = 0
negative z-score
no activity pattern available
Ratio
-log(p-value)
EIF2 Signaling
Regulation of eIF4 and p70S6K Signaling
mTOR Signaling
Ratio
C
-log(p-value)
Integrin Signaling
Cdc42 Signaling
Regulation of Actin-based Motility by Rho
Ratio
D
CXCR4+ memory CD4+ T cells
CXCR4- memory CD4+ T cells
FOS
CCR7
SOCS3
MYC
PDE3B
SOCS1
ID3
CISH
SELL
BIRC3
PASK
NFKB1
ABLIM1
IFI44L
HSPD1
AKT3
MX2
IFNAR1
NFKB2
NFKBIA
PDCD4
PIK3R1
CYTIP
relative expression
RA1 RA2 RA3 RA4 RA5 RA1 RA2 RA3 RA4 RA5

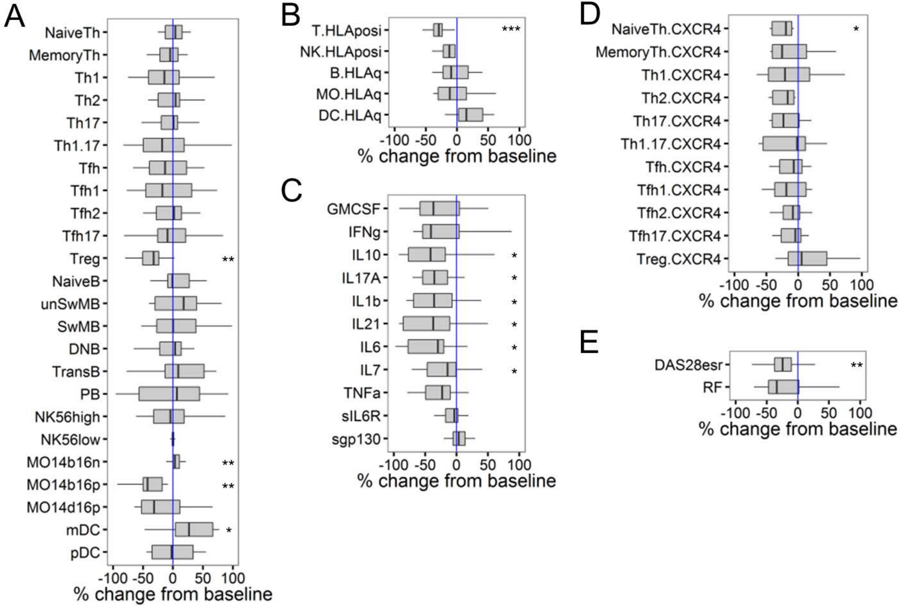

A
NaiveTh
MemoryTh
Th1
Th2
Th17
Th1.17
Tfh
Tfh1
Tfh2
Tfh17
Treg
**
NaiveB
unSwMB
SwMB
DNB
TransB
PB
NK56high
NK56low
MO14b16n
**
MO14b16p
**
MO14d16p
mDC
*
pDC
-100 -50 0 50 100
% change from baseline
B
T.HLAposi
***
NK.HLAposi
B.HLAq
MO.HLAq
DC.HLAq
-100 -50 0 50 100
% change from baseline
C
GMCSF
IFNg
IL10
*
IL17A
*
IL1b
*
IL21
*
IL6
*
IL7
*
TNFa
sIL6R
sgp130
-100 -50 0 50 100
% change from baseline
D
NaiveTh.CXCR4
*
MemoryTh.CXCR4
Th1.CXCR4
Th2.CXCR4
Th17.CXCR4
Th1.17.CXCR4
Tfh.CXCR4
Tfh1.CXCR4
Tfh2.CXCR4
Tfh17.CXCR4
Treg.CXCR4
-100 -50 0 50 100
% change from baseline
E
DAS28esr
**
RF
-100 -50 0 50 100
% change from baseline
